# An Ultra-microporous Carbon Material Boosting Integrated Capacitance for Cellulose-Based Supercapacitors

**DOI:** 10.1007/s40820-020-0393-7

**Published:** 2020-02-24

**Authors:** Chenfeng Ding, Tianyi Liu, Xiaodong Yan, Lingbo Huang, Seungkon Ryu, Jinle Lan, Yunhua Yu, Wei-Hong Zhong, Xiaoping Yang

**Affiliations:** 1 State Key Laboratory of Organic-Inorganic Composites, Beijing University of Chemical Technology, Beijing, 100029 People’s Republic of China; 2 Key Laboratory of Synthetic and Biological Colloids, Ministry of Education, School of Chemical and Material Engineering, Jiangnan University, Wuxi, 214122 Jiangsu People’s Republic of China; 3 Institute of Carbon Tech., Jeonju University, Jeonju, 55069 South Korea; 4 School of Mechanical and Material Engineering, Washington State University, Pullman, 99163 USA

**Keywords:** Integrated capacitance, Bacterial cellulose, Microporous carbon, Heteroatom doping, Supercapacitors

## Abstract

A breakthrough in advancing power density and stability of carbon-based supercapacitors is trapped by inefficient pore structures of electrode materials. Herein, an ultra-microporous carbon with ultrahigh integrated capacitance fabricated via one-step carbonization/activation of dense bacterial cellulose (BC) precursor followed by nitrogen/sulfur dual doping is reported. The microporous carbon possesses highly concentrated micropores (~ 2 nm) and a considerable amount of sub-micropores (< 1 nm). The unique porous structure provides high specific surface area (1554 m^2^ g^−1^) and packing density (1.18 g cm^−3^). The synergistic effects from the particular porous structure and optimal doping effectively enhance ion storage and ion/electron transport. As a result, the remarkable specific capacitances, including ultrahigh gravimetric and volumetric capacitances (430 F g^−1^ and 507 F cm^−3^ at 0.5 A g^−1^), and excellent cycling and rate stability even at a high current density of 10 A g^−1^ (327 F g^−1^ and 385 F cm^−3^) are realized. Via compositing the porous carbon and BC skeleton, a robust all-solid-state cellulose-based supercapacitor presents super high areal energy density (~ 0.77 mWh cm^−2^), volumetric energy density (~ 17.8 W L^−1^), and excellent cyclic stability.

**Electronic supplementary material:**

The online version of this article (10.1007/s40820-020-0393-7) contains supplementary material, which is available to authorized users.

## Highlights

- An ultra-microporous carbon material simultaneously with high specific surface area (1554 m^2^ g^−1^) and packing density (1.18 g cm^−3^) is designed and fabricated.
- The resulting carbon material integrates the high gravimetric and volumetric capacitance (430 F g^−1^ and 507 F cm^−3^ at 0.5 A g^−1^) and thereof provides the robust all-solid-state cellulose supercapacitor with high areal and volumetric density.

**Electronic supplementary material:**

The online version of this article (10.1007/s40820-020-0393-7) contains supplementary material, which is available to authorized users.

## Graphical abstract

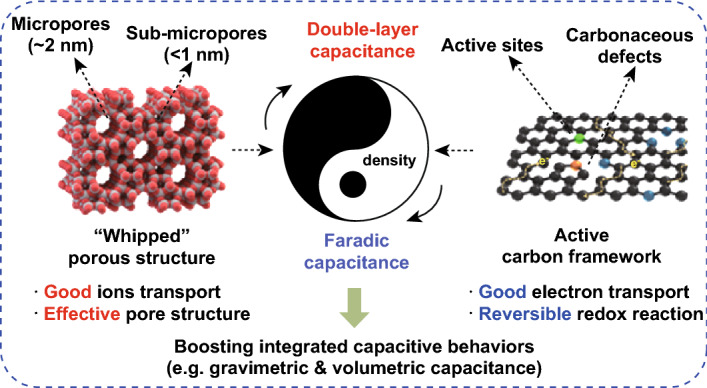

## Introduction

With the booming development of electric transportation and electronics, there are increasing demands for sustainable and renewable energy conversion and storage devices [1]. Supercapacitors are widely recognized as one type of the most promising energy storage systems (EES) due to competitive advantages, including high power density, long cycle life, and low maintenance cost [2]. However, the low energy density has dramatically limited the development and practical application of supercapacitors [3]. Electrode materials, as the essential capacitive component, play a vital role in the achievement of high-energy-density supercapacitors. Faced with challenges of complicated applications, the next-generation electrode materials with excellent integrated capacitive performances including high gravimetric, volumetric, and areal capacitance are much demanded.

Over the past few years, numerous efforts have been undertaken to achieve the excellent gravimetric and volumetric performance of pseudocapacitive electrode materials including transition metals [4], conducting polymers [5], pseudocapacitive 2D materials [6], and their composites [7]. In the reported studies, the pseudocapacitive electrode materials with high gravimetric and volumetric capacitance were achieved. However, the insufficient rate and cyclic stability still restrict their further enhancement for the performance of supercapacitors due to the repetitive material swelling/shrinkage of these materials during the charge/discharge processes [8, 9]. Compared with transition metals and conductive polymer materials, carbon materials are considered as more promising electrode materials for ultra-stable and high-performance supercapacitors due to the tunable packing structure, excellent doping modification, and good electrical conductivity [2]. Thus far, fossil-based carbon materials such as graphene [10], carbon nanotubes [11], metal-organic frameworks [12], coal-based activated carbons [13], and polymer-based carbons [14] have been intensively studied. The resulting supercapacitors have demonstrated to simultaneously possess satisfactory gravimetric and volumetric capacitance (ca. 300 F g^−1^ and 400 F cm^−3^). However, as the high-dense packing structures and nonporous characteristics of these carbon materials lack of pore paths for ion diffusion and transportation, high-power performance and cycling stability were sacrificed [15]. Meanwhile, costly nanotechnologies, complicated production procedures, and/or inevitable harmful by-products during the fabrication process are still critical challenges for broad applications of supercapacitors. Therefore, there is still a need for efforts on developing low-cost, sustainable, and stable electrode materials with further improved integrated capacitance for advanced supercapacitors.

As the most promising alternatives to fossil-based carbon materials, biomass-based carbon materials exhibit great prospects for practical industrialization [16]. Thus far, various high-performance biomass-based carbon materials have been reported, such as those derived from bone [17], fungus [18], gelatin [19], bamboo [20], bacterial cellulose [21], and lignocellulose [22]. Among various biomass precursors, cellulose and its derivatives show very promisingly due to their unique nanofibrous structures with high aspect ratios. Besides, the 3D nanofibrous structure of cellulose-derived carbons has been demonstrated to enable excellent mass transport and abundant active sites for the ionic transport and electronic conduction, and then potentially increase the rate and cycling stability [23]. Thus far, various high-performance cellulose-based carbon materials with high gravimetric performance have been reported [24, 25]. However, owing to the limited microporous surface area (*V*_micro_/*V*_total_ < 70%) and low packing density (< 1.0 g cm^−3^), the volumetric capacitance of biomass-derived carbon materials is still less than 500 F cm^−3^ [16, 26]. Furthermore, the reported improvement strategies, such as designing nonporous structure and heteroatom modification, just led to moderate the rate stability and power density of supercapacitors due to the poor ionic transport and electronic conduction. Therefore, to resolve these issues, more critical efforts on carbon materials with special porous structures that can realize good sustainability, low cost, high integrated capacitance, and high-power stability are highly needed.

Herein, we report a unique microporous carbon is achieved via a one-step carbonization/activation of dense bacterial cellulose nanofibers (BC) and then nitrogen/sulfur (N/S) dual doping. The microporous carbon exhibits concentrated micropores (~ 2 nm) notably including numerous sub-micropores (< 1 nm), leading to simultaneously high specific surface area and high packing density. Benefiting from the synergetic effects derived from the porous structure and effective N/S dual doping, the resulting u-MPC presents remarkable gravimetric and volumetric capacitance, excellent high-power rate and cycling stability. As a result, the assembled symmetric supercapacitor via compositing the “whipped” porous carbon and BC nanofibers exhibits robust mechanical performance, high area and volumetric energy density, and excellent cycling stability at the same time. This work provides new insights on porous carbon materials with high integrated capacitance for high-performance and mechanical supercapacitors.

## Experimental

### Materials

Bacterial cellulose (industrial grade product derived from coconut shells, Hainan Laize Biochemical Co.) was used as a carbon precursor. The potassium hydroxide (KOH) and thiourea (CN_2_H_4_S) acted as activated agent and nitrogen and sulfur dopant source, which was supplied by Beijing Tongguang Chemical Research Institute, China. Conductive graphite as a conductive agent was provided by Alfa Aesar, China. Polyvinylidene fluoride and *N*-methyl-pyrrolidone used for electrochemical testing were supplied by Aladdin, China. All other chemical reagents were analytical grades.

### Preparation of u-MPC

Preparation of microporous carbon (MPC): The as-received industrial BC pellicles were washed with deionized water and then cut into rectangular slices (5.0 × 5.0 × 0.5 cm^3^, length × width × thickness). The BC slices were immersed in 1 M KOH for 24 h at room temperature, and frozen in liquid nitrogen (− 196 °C) and then freeze-dried. The dried slices were compressed under 50 MPa into thick slices. The thick slices were heated under N_2_ flow in a carbonization furnace with 5 °C min^−1^ to the final temperature in the range of 700–900 °C and held for 2 h. After cooling, the carbonized carbonaceous slices were washed with 1 M HCl and deionized water to neutral and then dried at the oven with 100 °C for 12 h. For comparison, pure BC porous carbon was prepared by following the same procedure without KOH immersion.

Preparation of u-MPC: The as-prepared MPC was mixed to thiourea (CN_2_H_4_S) with a weight ratio of 1:1 by ball milling (400 r min^−1^). The mixture was heated under N_2_ flow in a carbonization furnace to 800 °C with a heating rate of 5 °C min^−1^ and held for 3 h. Then, carbonaceous materials were washed by deionized water to neutral and dried at the oven with 100 °C for 12 h to obtain u-MPC.

### Structure and Surface Chemistry Analysis

The morphologies of the samples were examined by a field emission scanning electron microscope (FESEM, Supra55, Carl Zeiss) and a high-resolution transmission electron microscope (HR-TEM, JEM-3010, JEOL). Structural analysis was performed by using a Raman spectroscopy (Ar laser (~ 3 mW), wavelength: 532 nm, RM2000, Renishaw) and a D8 Advance diffractometer (Bruker) with a Cu Kα source. Nitrogen adsorption/desorption isotherms were collected at 77 K on a Micromeritics ASAP 2020 instrument. The specific surface area (*S*_BET_) was obtained by the Brunauer–Emmett–Teller (BET) method. The micropore surface area (*S*_mic_) and micropore volume (*V*_mic_) were obtained from the t-Plot method. The summation of mesopores and macropores area was obtained from the deduction. The total pore volume (*V*_t_) was obtained from single-point adsorption (SPD), and pore size distribution was obtained from nonlocal density functional theory (NLDFT) method. The tap densities of the u-MPC powders were measured as follows: A certain quantity of powder was added to a dry quartz tube, which was then vacuumed until the volume of the powders did not change to measure the volume of the tapped powder. Then, the mass and the measured volume of the tapped powder were used to calculate its tap density.

### Electrochemical Measurements

To prepare the electrodes of the three-electrode system: A mixture of the sample and polyvinylidene fluoride binder with the weight percent ratio of 9:1 was dispersed in *N*-methyl-pyrrolidone, and then, the slurry was coated on the platinum current collectors. Afterward, the electrodes were dried in a vacuum oven at 120 °C for 24 h. The loading mass of the active materials on each platinum plate was about 2.5 mg cm^−2^. Before testing, the working electrodes were immersed in the H_2_SO_4_ electrolytes for 10 h to improve the wettability between electrode and electrolyte. The electrochemical measurements were separately carried out in a three-electrode system in H_2_SO_4_ electrolyte. The Ag/AgCl electrode and a slice of the platinum plate were used as the reference electrode and the counter electrode, respectively.

To prepare u-MPC/BC electrodes for symmetric devices: Industrial BC slices (2 × 2 cm^2^) were physically crushed and purified by KOH aqueous solution at 80 °C for 8 h. Then, the BC slurry was washed by deionized water to neutral and suspended in deionized water. The activated materials (u-MPC-800) were mixed with conductive graphite by ball milling (400 r min^−1^) for 2 h resulting in a solid content weight ratio of 1:1. Then, the mixture of u-MPC and conductive graphite was added into BC suspension with the solid content weight ratio of 3:1. The u-MPC/BC electrodes were prepared by vacuum filtration and dried by freeze-drying for 12 h. The loading mass of active materials on the electrodes was about 5 mg cm^−2^. Finally, before the assembling into symmetric devices, the electrodes were compressed under 20 MPa and immersed in 1 M H_2_SO_4_ for 2 h. The BC slices adsorbed 1 M H_2_SO_4_ electrolyte worked as gel electrolyte for symmetric supercapacitors.

The potential window in acidic electrolyte was between − 0.1 and 0.9 V. Cyclic voltammetry (CV, at various scan rates from 10 to 100 mV s^−1^) and electrochemical impedance spectroscopy (EIS, frequency from 10 Hz to 100 kHz) measurements were carried out on an electrochemical workstation (Autolab PGSTAT 302 N, Metrohm, Netherlands). The galvanostatic charge/discharge processes were conducted on a LAND CT2001A battery tester (China) at the current density from 0.5 to 20 A g^−1^. The gravimetric specific capacitance *C*_g_ (F g^−1^) in the three-electrode system was calculated based on Eq. (1):

1 $$C_{\text{g}} = I\Delta t/m\Delta V$$

where *m* (g) is the mass of active material on working electrode, *I* (A) is the discharge current, *∆t* (s) is the discharge time, and *∆V* (V) is the potential change including the voltage drop within *∆t*, respectively.

The volumetric specific capacitance *C*_v_ (F cm^−3^) in the three-electrode system was calculated based on Eq. (2):

2 $$C_{\text{v}} = C_{\text{g}} \times \rho_{\text{v}}$$

where *ρ*_v_ (g cm^−3^) is the packing density. The gravimetric specific capacitance *C*_g_ (F g^−1^), areal specific capacitance *C*_a_ (mF cm^−2^), gravimetric energy density *E*_g_ (Wh kg^−1^) and power density (W kg^−1^), and areal energy density *E*_a_ (µWh cm^−2^) and power density *P*_a_ (mW cm^−2^) in the two-electrode system were calculated based on Eqs. (3)–(10):

3 $$C_{\text{g}} = I \times \Delta t/M \times \Delta V$$

4 $$C_{\text{a}} = C_{\text{g}} \times \rho_{\text{a}}$$

5 $$E_{\text{g}} = C_{\text{g}} \times \Delta V^{2} /2$$

6 $$P_{\text{g}} = \, 3600 \times \, E_{\text{g}} /\Delta t$$

7 $$E_{\text{a}} = E_{\text{g}} \times \rho_{\text{a}}$$

8 $$P_{\text{a}} = P_{\text{g}} \times \rho_{\text{a}}$$

9 $$E_{\text{v}} = E_{\text{g}} \times \rho_{\text{v}}$$

10 $$P_{\text{v}} = P_{\text{g}} \times \rho_{\text{v}}$$

where *M* (g) is the total mass of the active materials on the two electrodes, *I* (A) is the discharge current, *∆t* is the discharge time, *∆V* (V) is the potential change including the voltage drop within *∆t*, and *ρ*_a_ (g cm^−2^) is the areal loading mass on the electrode.

## Results and Discussion

### Effects of Carbonization Temperature on the MPC Structures

**Fig. 1:**
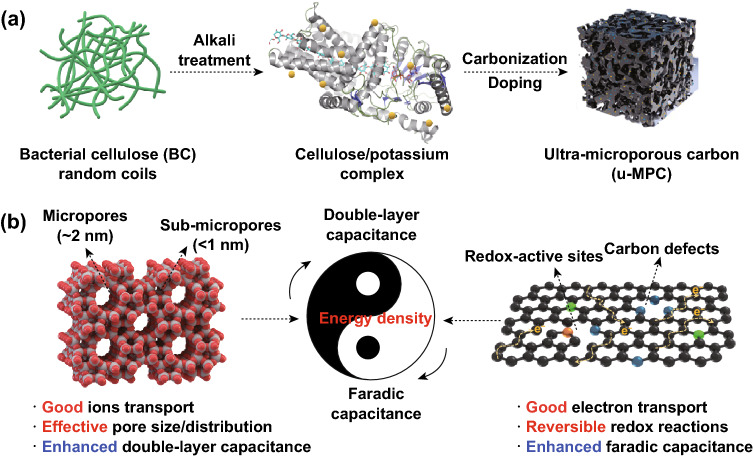
Fabrication of u-MPC and enhancement mechanism on energy density. **a** Schematic illustration for the fabrication of u-MPC. **b** Schematic diagram of the enhancements of pore structure and carbonaceous constituent to energy density

In this work, a unique porous carbon is designed by optimization of pore structure and regulation of carbonaceous constituent to achieve high integrated capacitance. As illustrated in Fig. 1a, the BC nanofibers are pretreated by KOH solution, which results in 3D ions–fiber complex rich in strong electrostatic interaction. The complex is converted into dense BC/KOH composite precursor by freeze-drying and then compression. After one-step carbonization/activation, the dense composite precursor in Fig. S1 is converted to porous carbon materials. The dense BC/KOH precursor exhibits good dispersion of K^+^, compact packing structure, and 3D nanofibrous network structure. These merits warrant the resulting porous carbon with a concentrated porous structure and compact structure. Furthermore, the heteroatom doping on carbon framework induces structural defects and redox sites to enhance the capacitive performance. The synergistic effects from the porous structure and optimal carbonaceous constituent in Fig. 1b boost the capacitive performance of carbon electrode material for advanced supercapacitors.

**Fig. 2:**
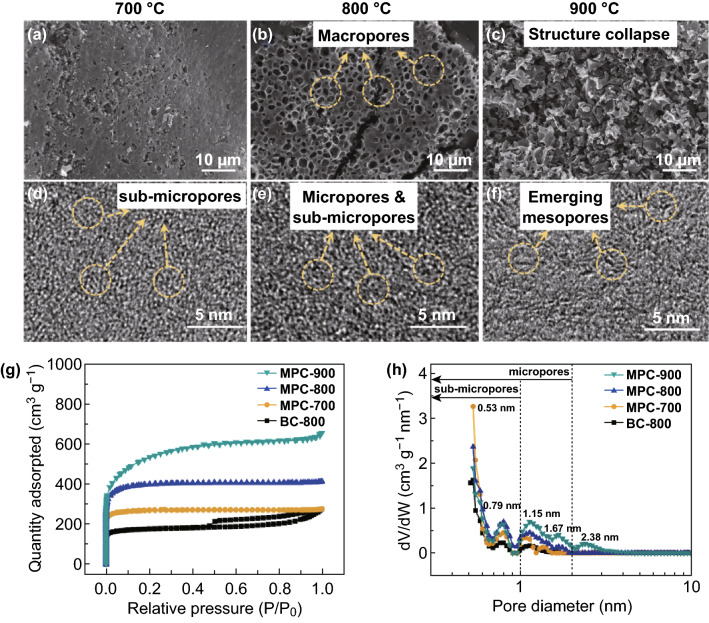
Morphologies and pore structure of the MPCs treated under various temperatures. SEM images of **a** MPC-700, **b** MPC-800, and **c** MPC-900. HR-TEM images of **d** MPC-700, **e** MPC-800, and **f** MPC-900. **g** Nitrogen adsorption–desorption isotherms. **h** Pore size distribution of BC-800 and MPCs series

Carbonization temperature is crucial to the pore structure and microstructure of carbon materials. Pore evolution in the MPC samples at various temperatures is shown in Fig. 2. With one-step carbonization/activation process, the dense nanofibrous precursor converts into porous carbon particles due to the fusion of nanofibers during carbonization. Specifically, compared with the MPC-700 in Fig. 2a, MPC-800 in Fig. 2b exhibits a honeycomb-like porous structure consisting of numerous macropores. Moreover, there are lots of interconnected pores on and inside macropore walls of the MPC-800 in Fig. S2, which are beneficial to the development of micropores and sub-micropores on the macropore wall. The pore structure is quite different from that of BC-800 in Fig. S3. However, increasing the temperature up to 900 °C, the porous structure in Fig. 2c is crumbled due to the overactivation.

The microstructure transformation under various carbonization temperatures is demonstrated by HR-TEM in Fig. 2d–f. Specifically, the MPC-700 in Fig. 2d presents the defective and disordered carbon structure with dominant sub-micropores. With higher carbonization temperature, the MPC-800 in Fig. 2e exhibits some partial stacking carbon layers and microporous structure. As the carbonization temperature up to 900 °C, MPC-900 in Fig. 2f shows more stacking carbon layers and even the generation of mesopores. Thus, the carbonaceous microstructure is mainly affected by carbonization temperature on two aspects: One is that carbonization temperature can affect the graphitization process. With the increase in carbonization temperature, there are more stacking carbon layers, which means a higher degree of graphitization. Another is that carbonization temperature influents the pore structure development by chemical and physical activation with KOH and its mid-products such as potassium carbonate (K_2_CO_3_), potassium oxide (K_2_O), and metallic potassium (K) as Eqs. (11)–(15):

11 $${\text{KOH}} + 2{\text{C}} \to {\text{K}} + {\text{H}}_{2} + {\text{K}}_{2} {\text{CO}}_{3}$$

12 $${\text{K}}_{2} {\text{CO}}_{3} \to {\text{K}}_{2} {\text{O}} + {\text{CO}}_{2}$$

13 $${\text{CO}}_{2} + {\text{C}} \to 2{\text{CO}}$$

14 $${\text{K}}_{2} {\text{CO}}_{3} + 2{\text{C}} \to 2{\text{K}} + 3{\text{CO}}\left( {{\text{over}}\;700\,^\circ {\text{C}}} \right)$$

15 $${\text{C}} + {\text{K}}_{2} {\text{O}} \to 2{\text{K}} + {\text{CO}}\left( {{\text{over}}\;700\,^\circ {\text{C}}} \right)$$

With the carbonization temperature under 700 °C, the pores are mainly developed through etching carbon framework by Eqs. (11)–(13). As illustrated in Eqs. (14) and (15), with the carbonization temperature over 700 °C, the generation of metallic K can result in the expansion of carbon lattice and develop pores [27]. Moreover, the metallic K can also convert into gasification K, which can increasingly generate the pore structure by gasification K and further chemical activations.

The pore evolutions are further demonstrated by nitrogen adsorption/desorption isotherms analysis. As shown in Fig. 2g, the adsorption/desorption curve of BC-800 exhibits a combined type-I/IV sorption isotherm with little knee and visible hysteresis loop, indicating low content of micropores and typical mesoporous structure. However, the adsorption/desorption curves of MPC series show typical type-I curves with a sharp nitrogen adsorption knees at a relative pressure below 0.05, demonstrating standard microporous structure [28]. Moreover, Table 1 exhibits the values of *V*_mic_/*V*_t_ higher than 50%, which shows MPC series are microporous carbon. Among these MPC series, MPC-800 presents some excellent properties including the highest microporous surface area (~ 1311.4 m^2^ g^−1^), microporous volume ratio (~ 84%), and consequently minimum average pore size (~ 2.36 nm). The high microporous surface area is significantly beneficial to the ELDC capacitance.

**Table 1:** Characterization of pores and specific capacitance of MPC series

Sample	*S*_BET_ (m^2^ g^−1^)	*S*_mic_ (m^2^ g^−1^)	*S*_ext_ (m^2^ g^−1^)	*V*_*t*_ (cm^3^ g^−1^)	*V*_mic_ (cm^3^ g^−1^)	*D*_ave_ (nm)
BC-800	676.7	565.2	111.5	0.44	0.23	2.58
MPC-700	1038.6	949.0	89.7	0.44	0.38	2.60
MPC-800	1554.5	1311.4	243.2	0.65	0.52	2.36
MPC-900	1980.2	876.6	1103.6	1.02	0.35	2.83

*S*_*BET*_ the specific surface area, *S*_*mic*_ the micropore specific surface area, *S*_*ext*_ the mesopore and the macropore specific surface area, *V*_*total*_ the total volume, *V*_*mic*_ the microporous volume, *D*_*ave*_ the average diameter of pores

The pore size distribution, which directly affects the utilization efficiency of surface area, is quite vital for the achievement of the high capacitance of carbon materials. The pore size of all MPCs in Fig. 2h is concentrated in micropores, which is consistent with the previous result of morphologies (see Fig. 2d–f). Specifically, MPC-900 presents boarder pore distribution ranging from 0.5 to 3.0 nm, demonstrating the appearance of several mesopores on the MPC-900. MPC-700 and MPC-800 show relatively centralized pore distribution from 0.5 to 2 nm, which mainly concentrates on four pore sizes such as 0.53, 0.79, 1.15, and 1.67 nm. Among these pores, sub-micropores with size below 1 nm significantly enhance the capacitive performance due to pore confinement effect [29, 30].

The graphitization crystal structure of the MPC series is investigated by the analysis of Raman spectroscopy and XRD spectra. As shown in Fig. S4a, the Raman spectra curves are fitted into five Gaussian regions, including *G* (at ~ 1580 cm^−1^, presenting in *sp*^2^ bonded graphitic carbons), D (at ~ 1350 cm^−1^, exhibiting *sp*^3^ defects), I (at ~ 1220 cm^−1^, attributed to the impurities or heteroatoms in the graphitic lattice), D’ and D’’ (at ~ 1620 and ~ 1490 cm^−1^, caused by the irregular *d*_002_ spacing and the defects in graphene layer stacking) [31]. The relationship between the carbonaceous structure and carbonization temperature is illustrated in Fig. S4b. The remarkable high contribution of D band demonstrates all MPC series are amorphous carbons, which is consistent with the previous HR-TEM results. As compared with other samples, MPC-800 exhibits the highest proportion of D’’, indicating that there are numerous defects in the graphitic layers. The defects in the graphitic layers are mainly attributed to the expansion and etching by metallic K. As carbonization temperature up to 900 °C, some defects in carbon framework are developed into micropores, which is consistent with the result in Fig. 2h. Moreover, the I band slightly decreases with the increase in carbonized temperature due to the formation of stable carbon framework. The carbon crystal structure is characterized by XRD analysis in Fig. S4c. Two broad peaks are located at about 22.8° and 43.8°, which are attributed to the (002) and (100) reflections of the crystal planes in carbon materials. The broad peaks demonstrate the amorphous feature of the MPC series, which is consistent well with the Raman analysis result and previous HR-TEM morphology results.

**Fig. 3:**
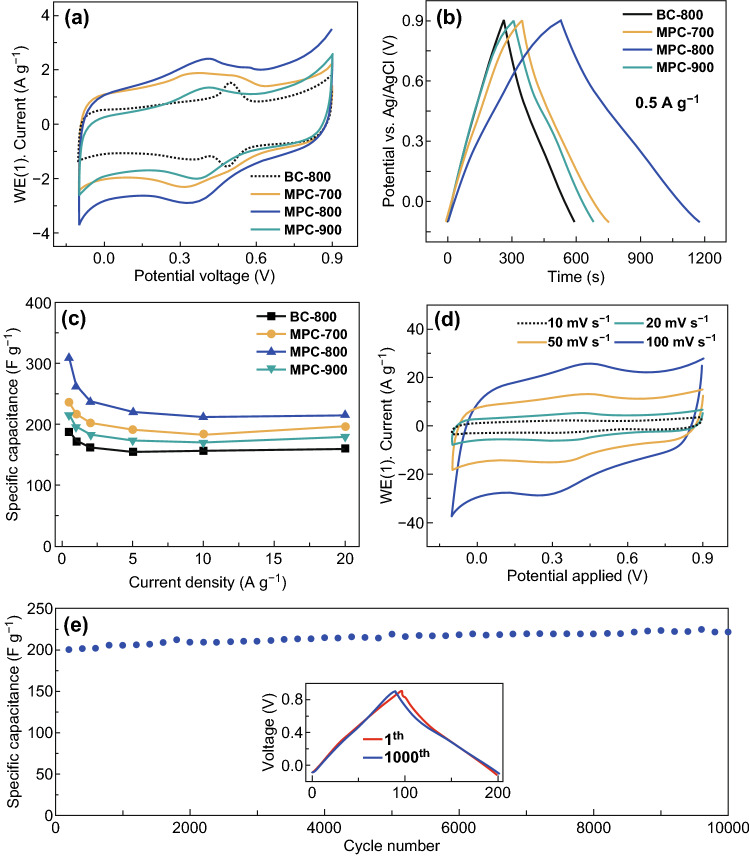
Electrochemical performance of the MPC series in a three-electrode system. **a** CV curves of MPC series at 10 mV s^−1^. **b** Charge/discharge curves at 0.5 A g^−1^. **c** Specific capacitance versus current density. **d** CV curves of MPC-800 at various scan rates (10–100 mV s^−1^). **e** Long-term cycle performance and Coulombic efficiency of MPC-800 at 10 A g^−1^ with charge/discharge curves before and after 10,000 cycles inset

To demonstrate the pore structure for improving electrochemical properties, the electrochemical behaviors of the MPC series are shown in Fig. 3. The CV curves in Fig. 3a exhibit a quasi-rectangular shape, indicating the predominant EDLC contribution. Specifically, CV curves show inconspicuous broad humps around 0.4 V, which are attributed to the reversible redox reactions of the inherent oxygen-containing functional groups [32]. The largest loop area of MPC-800 indicates the highest capacitive performance. The galvanostatic charge/discharge curves in Fig. 3b exhibit good symmetricity, indicating high reversibility of charge/discharge processes. The excellent capacitive performance of MPC-800 is attributed to the combined effects of high micropore volume and optimal pore size distribution. The rate performance, as a quite important factor for power density, is shown in Fig. 3c, d. The specific capacitance of BC-800, MPC-700, MPC-800, and MPC-900 is calculated to be 188, 235, 318, and 219 F g^−1^ at 0.5 A g^−1^ in Fig. 3c, respectively. Even at a high current density of 20 A g^−1^, MPC-800 still retains high specific capacitance (~ 219 F g^−1^), corresponding to the high capacitance retention (~ 68%). The excellent capacitive performance of MPC-800 is attributed to the high microporous surface area and centralized pore distribution. Furthermore, the CV curves varying from 10 to 100 mV s^−1^ are plotted in Fig. 3d to demonstrate the rate stability of MPC-800. Even the scanning rate up to 100 mV s^−1^, MPC-800 remains the initial rectangular-shaped curves, indicating excellent rate stability. The long-term stability at 10 A g^−1^ is tested for 10,000 charge/discharge cycles in Fig. 3e. Interestingly, the capacitance retention after long-term cycling is more than 100%. This is attributed to the slow activation of carbon layers during cycling, which leads to generating more active sites for ion storage. The initial and final charge/discharge curves are almost overlapped to demonstrate good long-term stability. The good cyclic stability is attributed to the stable pore structure that provides excellent ion transport and electron conduction.

### Influence of N/S Doping on the MPCs

It is known that high ion accessible surface area, efficient redox reactions, and high packing density are in the tug-of-war relationship. The simultaneous achievement of these performances is quite vital for the significant improvement of integrated capacitance. To further promote the capacitive performance, the N and S atoms are introduced into the carbon framework to optimize the pore structure and electronic feature. The heteroatom composition in the carbon framework is revealed by XPS spectra analysis in Table S1. The u-MPC displays higher content of nitrogen (~ 5.2%) and sulfur atoms (~ 1.2%) in the carbonaceous structure than that of the counterparts. As shown in Fig. S5a, the high-resolution spectra of S 2p present three major S groups at binding energies of ~ 164, ~ 165.2, and ~ 169.2 eV, respectively [33]. The two prominent peaks at ~ 164 and ~ 165.2 eV correspond to S 2p_3/2_ (S-1) and S 2p_1/2_ (S-2), which are the C–S–C covalent bonds of the thiophene-S caused by spin-orbit coupling. The broad peak of S-3 at ~ 169.2 eV is appointed to the oxidized sulfur of C–SOx–C bond [34]. The S atoms in the carbon framework can enhance electrical conductivity and induce structural defects on the carbon framework, which are beneficial to the improvement of capacitive behavior and electrochemical stability. Meanwhile, owing to the large S lone pairs, the enhanced polarizability further favors the interaction with active oxygen atoms, which improve the ability to capture ions and then initial pseudocapacitive behaviors [35]. The deconvolution of N 1 s peak in Fig. S5b exhibits three significant peaks, i.e., pyridinic N (N-6 at ~ 398.2 eV), quaternary N (N–Q at ~ 400.8 eV), and oxides of nitrogen (N–X at ~ 402.4 eV) [36, 37]. The compositions of three nitrogen bonding are 25.4%, 38.2%, and 36.4%, respectively. The N-6 groups offer electrons for conjugation with the π-conjugated rings, which provide the electron donor characteristics to carbonaceous structure [38]. And it is believed that N–X groups are quite beneficial to the reversible Faradic reactions, which further enhance the capacitance. Furthermore, the EDX analysis in Fig. S5c–e exhibits the good distribution of S and N atoms, which is favorable to the reversible Faradic reactions in the whole carbon framework.

**Fig. 4:**
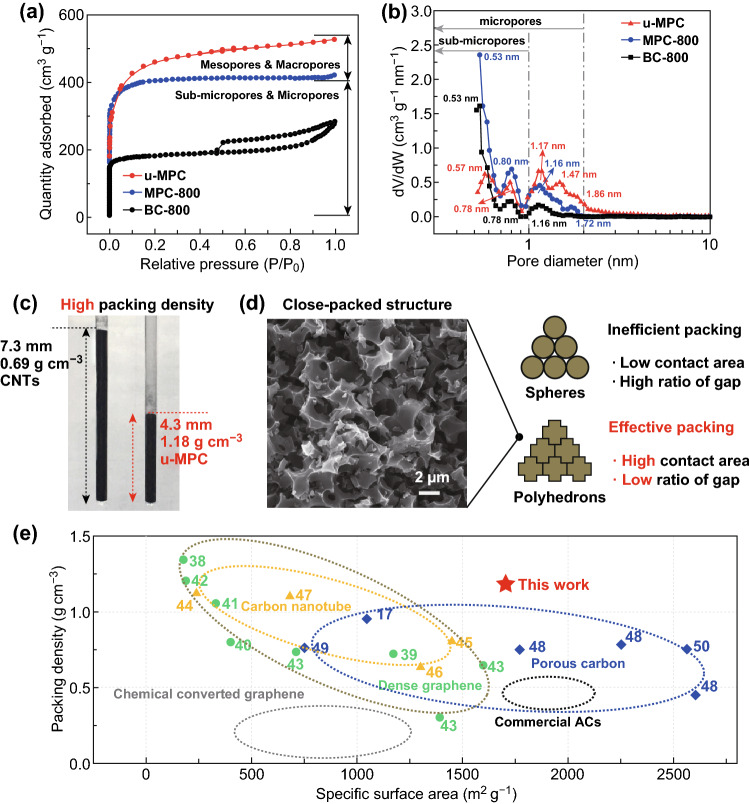
Pore parameter and packing density of u-MPC as compared with counterparts. **a** Nitrogen adsorption/desorption isotherms. **b** Pore size distribution. **c** Packing density as compared with CNTs. **d** Morphology of carbon particles and schematic illustration of packing structure with various particles. **e** Packing density versus specific surface area as compared with other work

The optimal ion accessible surface area provides several ionic conduction pathways and active sites for ion storage to enhance the electrochemical performance. The pore structure of u-MPC is investigated by nitrogen adsorption/desorption analysis. The u-MPC exhibits a combined type-I/IV sorption isotherm in Fig. 4a, indicating the generation of mesopores. Moreover, the pore size distribution in Fig. 4b further reveals the influence of N/S doping on pore structure. The surface heteroatoms such as nitrogen and sulfur atoms are affecting the pyrolysis behavior of the underlying carbon at higher temperatures, particularly in the formation and/or retention of micropores [39]. Via induction effects of N/S atoms, the surface area increases to 1704 m^2^ g^−1^, and some sub-micropores convert to micropores between 1 and 2 nm resulting in a more centralized average pore size of 2.2 nm in Table S2. Accordingly, the u-MPC displays an excellent microporous surface area ratio of 86%, which is even higher than MPC-800. Furthermore, the u-MPC exhibits the larger pore volume of 0.81 cm^3^/g than that of MPC-800 (0.65 cm^3^ g^−1^) and BC-800 (0.44 cm^3^ g^−1^). Thus, the introduction of N and S into carbon framework improves the pore structure on two aspects: One is that it can maintain original excellent pore structure and further develop more defects on carbon framework; another is that it can further centralize the pore size. This improvement in pore structure is not only beneficial to the electrolyte infiltration and ion transport but also further enhanced capacitive performance.

In addition, packing density is a quite crucial property that can directly affect the practical electrochemical performance such as volumetric capacitance and areal capacitance. As shown in Figs. 4d and S6a, u-MPC particles present the unique polyhedral shape that stacks tightly with an average particle size (~ 2.4 µm). As compared with other particles such as sphere particles in Fig. 4d, the polyhedral particles are more conducive to the mutual accumulation [40]. The sectional morphology of u-MPC carbon particles in Fig. S6b exhibits solid characters, which is attributed to the highly concentrated microporous structure. Thus, the u-MPC shows a high packing density of 1.18 g cm^−3^ in Fig. 4c as compared with CNT powder of 0.69 g cm^−3^. Moreover, the packing density versus specific surface area of u-MPC is plotted in Fig. 4e as compared with other reported electrode materials such as graphene [41–46], carbon nanotube [47–50], porous carbon [18, 51–53], commercial activated carbon and chemical converted graphene [30]. Compared with other carbon materials, u-MPC simultaneously presents high packing density and high specific surface area, which are advantageous for the achievement of highly dense electrodes.

**Fig. 5:**
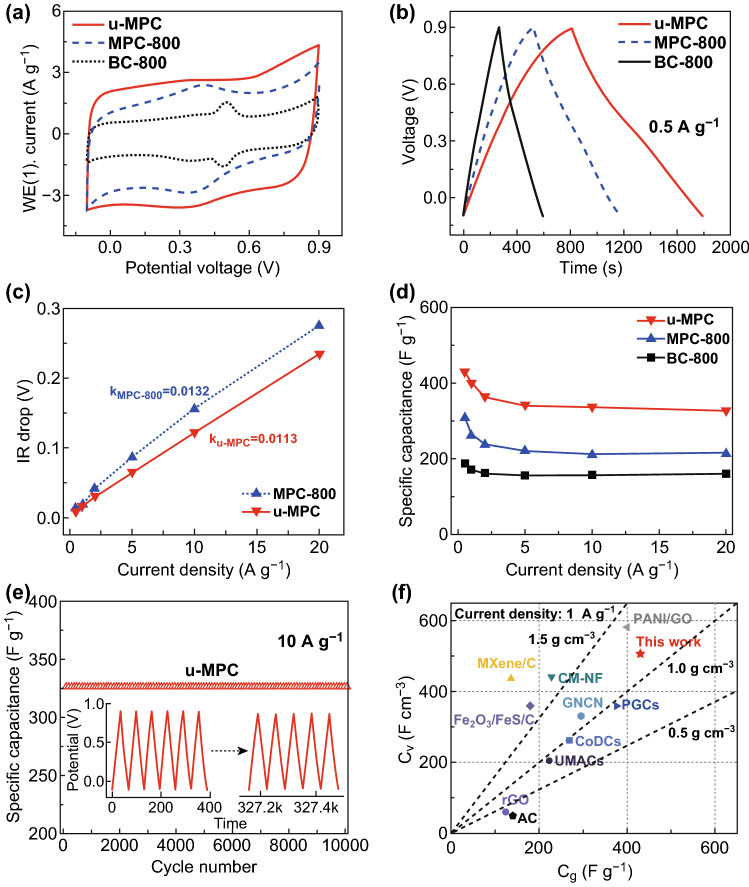
Electrochemical performance of u-MPC as compared with counterparts. **a** CV curves at 10 mV s^−1^. **b** Charge/discharge curves at 0.5 A g^−1^. **c** Plots of IR drop versus current densities. **d** Specific capacitance versus current density (0.5–20 A g^−1^). **e** Long-term cycling performance at 10 A g^−1^. **f** Volumetric and gravimetric capacitance as compared with other reported work

The electrochemical performance of u-MPC as compared with the counterparts is revealed in Fig. 5. Firstly, the CV curve in Fig. 5a exhibits a unique quasi-rectangular shape with board hump, indicating the coexistence of EDLC and pseudocapacitance in u-MPC that is attributed to the synergetic effect from nitrogen and sulfur atoms in the carbonaceous structure [34, 54, 55]. And u-MPC displays the largest loop area among these CV curves, indicating excellent capacitive behavior. The charge/discharge curves in Fig. 5b exhibit high symmetry and a little distortion during the charge/discharge process owing to reversible redox reactions happen during the charge/discharge process. The electron and ion transfer of u-MPC is investigated by plots of IR drop versus current densities (0.5–20 A g^−1^) in Fig. 5c. The slope of u-MPC line is calculated to 0.011, which is lower than that of MPC-800 (0.013), indicating good electron and ion transportation. According to the impedance curve in Fig. S7, the u-MPC presents lower bulk and interfacial resistance, demonstrating excellent electronic/ionic transportation and good interfacial compatibility with electrolyte. As illustrated in Fig. 5d, u-MPC exhibits high gravimetric capacitance of 430 F g^−1^ at 0.5 A g^−1^ and still retains high specific capacitance of 326 F g^−1^ at 20 A g^−1^ corresponding to retention ratio of 76%. Furthermore, via quantitatively evaluated by Dunn’s method [56], the capacitive and dissuasive contribution to total stored charge are further exhibited in Fig. S8. The diffusion contribution of the u-MPC is ~ 21.6% at the low current density of 10 mV/s, which is much higher than the 17.4% values for MPC-800. The improved diffusion contribution is attributed to the introduction of nitrogen and sulfur atoms into carbonaceous constituent, which is favorable to the reversible faradic reaction at low current densities [21, 35, 57]. At the high current density, the electric double-layer capacitive contribution is dominant part in the whole capacity [58]. Benefiting from the improved pore structure, even at 100 mV/s, the u-MPC exhibits the capacitive contribution of 97%, which is higher than that of MPC-800 (~ 95%). The excellent capacitive and rate performance of the u-MPC is attributed to synergetic effects from the concentrated pore structure and the optimal heteroatoms modification on the carbon framework. The electrochemical and structural stability is characterized by cyclic charge/discharge in Fig. 5e. The u-MPC presents stable long-term cycling even at 10 A g^−1^ with the capacitance retention of almost 100% after 10,000 cycles. As compared with the initial charge/discharge curves, the final charge/discharge curves in Fig. 5e inset show no visible difference, indicating the stable charge/discharge process during long cycling. More significantly, as compared with other reported electrode materials in Fig. 5f, the u-MPC simultaneously exhibits higher gravimetric and volumetric capacitance. The simultaneous achievement of gravimetric and volumetric capacitance is highly beneficial to the high energy density for advanced supercapacitors.

### Characterization of the All-Solid-State Cellulose-Based Supercapacitors

**Fig. 6:**
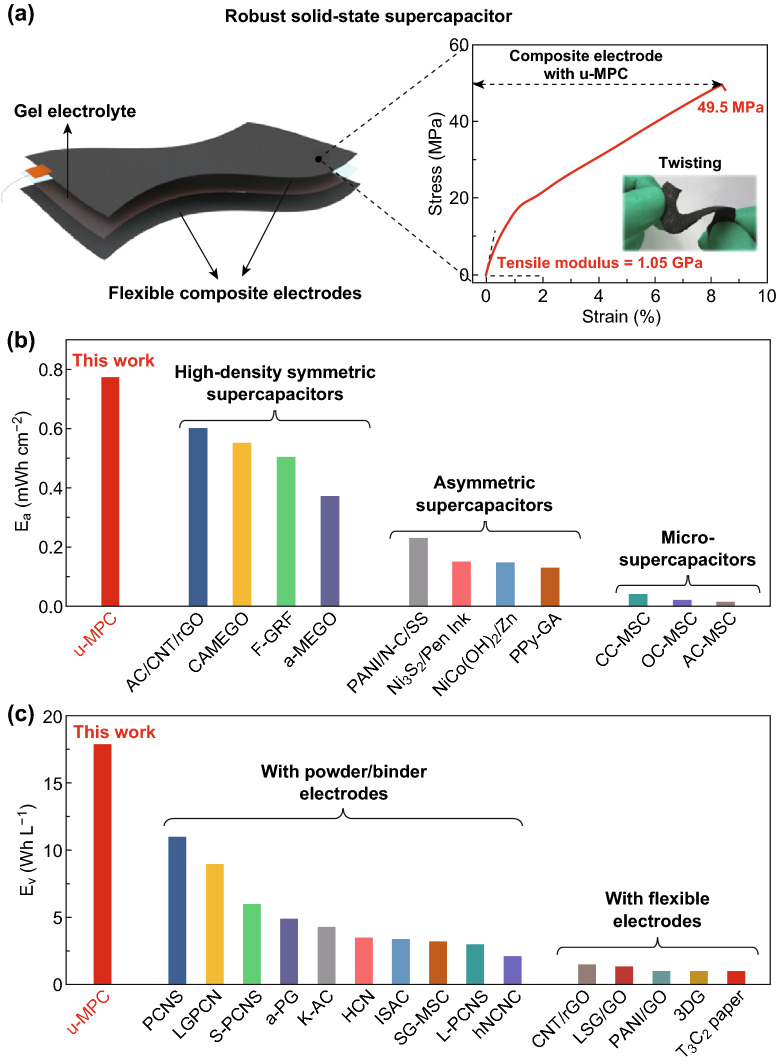
Performance of robust symmetric supercapacitors. **a** Schematic structure of robust symmetric supercapacitor and mechanical performance of composite electrode with a photograph of double-twisting composite electrode inset. Comparison of areal energy density (**b**) and volumetric energy density (**c**) with other reported work (*AC/CNT/rGO* activated carbon/carbon nanotube/reduced graphene oxide, *CAMEGO* compressed activated microwave-expanded graphene oxide, *F-GRF* multilayer-folded graphene ribbon film, *a-MEGO* activated microwave-expanded graphene oxide, *PANI/N-C/SS* polyaniline/nitrogen-doped carbon coated SS mesh, *Ni*_*3*_*S*_*2*_*/Pen ink* Ni_3_S_2_ nanorods/Pen ink, *NiCo(OH)*_*2*_*/Zn* NiCo(OH)_2_/Zn anode, *PPy-GA* polypyrrole–graphene aerogel, *CC-MSC* carbide-derived carbon-micro-supercapacitor, *OC-MSC* onion-like carbon-micro-supercapacitor, *AC-MSC* activated carbon-micro-supercapacitor; porous carbon nanosheet, *LGPCN* large-size graphene-like porous carbon nanosheet, *S-PCNS* scaffolding porous carbon nanosheet, *a-PG* activated PU-GO, *K-AC* KOH-activated carbon, *HCN* hollow carbon nanosphere, *ISAC* immense surface area carbon, *SG-MSC* sulfur-doped graphene micro-supercapacitor, *L-PCNS* layered porous carbon nanosheets, *hNCNC* hierarchical nitrogen-doped carbon nanocage, *LSG/GO* laser scribing graphene, *PANI/GO* polyaniline/graphene oxide, *3DG* three-dimensional graphene, *Ti*_*3*_*C*_*2*_ *paper* Ti_3_C_2_ composite paper)

Assembling into full supercapacitors is a crucial way to testify the performance and potential of electrode materials in practical applications. As illustrated in Fig. 6a, the robust electrodes via compositing u-MPC and BC nanofibers are assembled into symmetric supercapacitor with BC gel electrolyte. Faced with challenges of complex mechanical working conditions, advanced supercapacitors are highly needed to exhibit good flexibility and even excellent mechanical performance. The robust composite electrode presents excellent flexibility and mechanical tolerance even under twisting shown as insert Fig. 6a. Furthermore, the mechanical strength of electrodes and gel electrolyte are characterized by a tensile test. According to the strain–stress curves in Fig. 6a, the robust composite electrodes exhibit their tensile strengths of 49.5 MPa and Young’s modulus of 1.05 GPa. The high mechanical strength was mainly contributed by two parts: One is that the continuous BC skeleton supports electrode and provides high mechanical strength to the composite electrode; the other one is that the strong interactions (e.g., hydrogen bonding and Van der Waals force) between carbon particles and nanofibrous skeleton further enhance the strength and modulus of the composite system.

The electrochemical performance of symmetric supercapacitor is characterized by galvanostatic charge/discharge test at different current density (0.1–2 A g^−1^) in Fig. S9a. The charge/discharge curves exhibit good symmetry and no visible voltage drop, which indicates good reversibility and stability of the assembled supercapacitor. As illustrated in Fig. S9b, the symmetric supercapacitor exhibits high capacitance of 111 F g^−1^ at 0.1 A g^−1^ with the active loading mass of 5 mg cm^−2^. Accordingly, the supercapacitor presents excellent energy density, including areal energy density (ca. 0.77 mWh cm^−12^) and volumetric energy density (ca. 17.9 Wh L^−1^) in Fig. S10. As compared with other reported supercapacitors, the resulting supercapacitor armed with u-MPC exhibits much higher energy density including both areal energy density [46, 59–68] and volumetric energy density [53, 59, 69–81] as shown in Fig. 6b, c. Moreover, the excellent long-term stability of the symmetric supercapacitor is demonstrated by the reversible charge/discharge process at even 1.5 mA cm^−2^ for 10,000 cycles in Fig. S11. The supercapacitor displays almost 96% capacitance retention with 100% Columbic efficiency even after 10,000 cycles. The impedance curves after 10,000 cycles in Fig. S12 present still low bulk resistance with higher interface resistance as compared with that before long-term cycling. The excellent electrochemical behavior of the supercapacitor is contributed from both excellent capacitive performance and high packing density of the unique porous carbon electrode material.

## Conclusions

In summary, an ultra-microporous carbon material (u-MPC) with ultrahigh integrated capacitance and super rate stability is reported. The unique w-MPC is fabricated via one-step carbonization/activation of a dense BC/KOH composite precursor and then nitrogen/sulfur doping. The unique structure of the resulting u-MPC includes concentrated micropores (~ 2 nm), especially with numerous sub-micropores (< 1 nm). Owing to the exceptional pore structure, the u-MPC simultaneously exhibits a high specific surface area (1554 m^2^/g) and high packing density (1.18 g cm^−3^). Benefiting from synergistic effects from the particular pore structure and effective dual doping, remarkable specific capacitance including gravimetric and volumetric capacitance (430 F g^−1^ and 507 F cm^−3^ at 0.5 A g^−1^), excellent rate capability (327 F g^−1^ and 385 F cm^−3^ at 20 A g^−1^ corresponding to retention of 76%), and excellent cycling stability at high current density (10 A g^−1^) even for 10,000 cycling times is achieved. More significantly, via compositing the u-MPC and BC nanofibers, a robust symmetric supercapacitor integrates high areal energy density (~ 0.77 mWh cm^−2^), volumetric energy density (~ 17.9 Wh L^−1^), and excellent mechanical strength (49.5 MPa), which is higher than other reported supercapacitors. In addition, the assembled supercapacitor also presents long-term cyclic stability even at 1.5 mA cm^−2^. This work aims to provide a new strategy to fabricate carbon materials with high integrated capacitance for advanced robust supercapacitors.

## Electronic Supplementary Material

Below is the link to the electronic supplementary material.

Supplementary material 1 (PDF 1302 kb)
