# Supplementary material for: An Ultra-microporous Carbon Material Boosting Integrated Capacitance for Cellulose-Based Supercapacitors
Source: Nanomicro Lett. 2020 Feb 24;12:63. doi: 10.1007/s40820-020-0393-7 (PMC7770663; doi:10.1007/s40820-020-0393-7)
Supplement: Supplementary file 1 — Supplementary material 1 (PDF 1302 kb) [file 40820_2020_393_MOESM1_ESM.pdf]

Supporting Information for

An Ultra-microporous Carbon Material Boosting Integrated Capacitance for Cellulose-based Supercapacitors

Chenfeng Ding[1, 4], Tianyi Liu[1], Xiaodong Yan[2], Lingbo Huang[1], Seungkon Ryu[3], Jinle Lan[1], Yunhua Yu[1, *], Wei-Hong Zhong[4, *], Xiaoping Yang[1]

[1]State Key Laboratory of Organic-Inorganic Composites, Beijing University of Chemical Technology, Beijing 100029, People's Republic of China

[2]Key Laboratory of Synthetic and Biological Colloids, Ministry of Education, School of Chemical and Material Engineering, Jiangnan University, Wuxi, Jiangsu 214122, People's Republic of China

[3]Institute of Carbon Tech., Jeonju University, Jeonju 55069, South Korea

[4]School of Mechanical and Material Engineering, Washington State University, Pullman 99163, USA

*Corresponding authors. Email: katie_zhong@wsu.edu (Wei-Hong Zhong); yuyh@mail.buct.edu.cn (Yunhua Yu)

Supplementary Figures and Tables

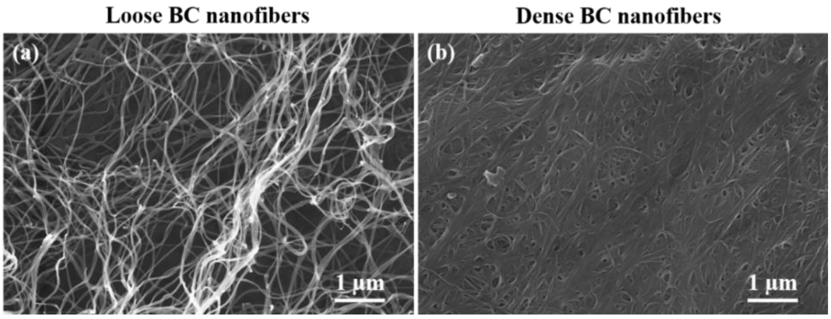

Fig. S1 a SEM image of loose BC nanofibers. **b** SEM image of dense BC nanofibers

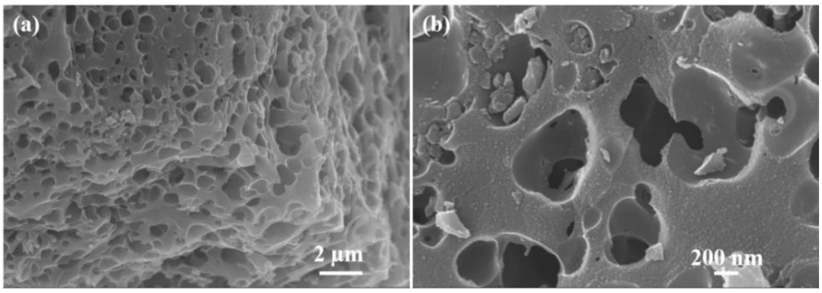

Fig. S2 a, b SEM images of MPC-800 porous structure and magnified surface morphology

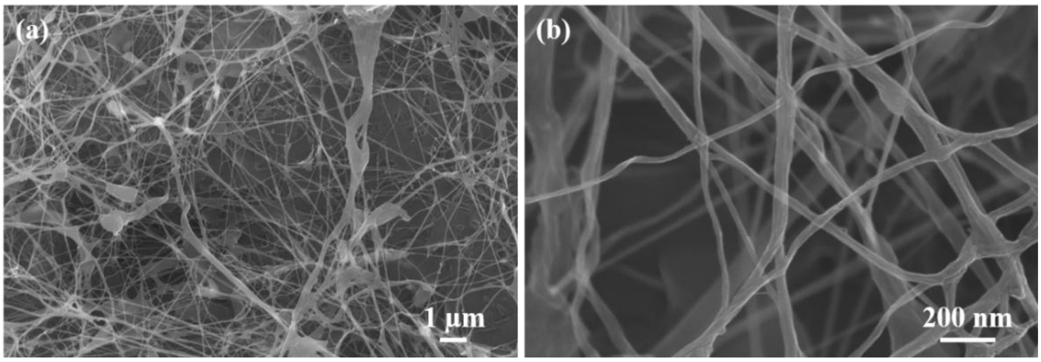

Fig. S3 a, b SEM images of BC-800 carbonized under 800 °C without activation

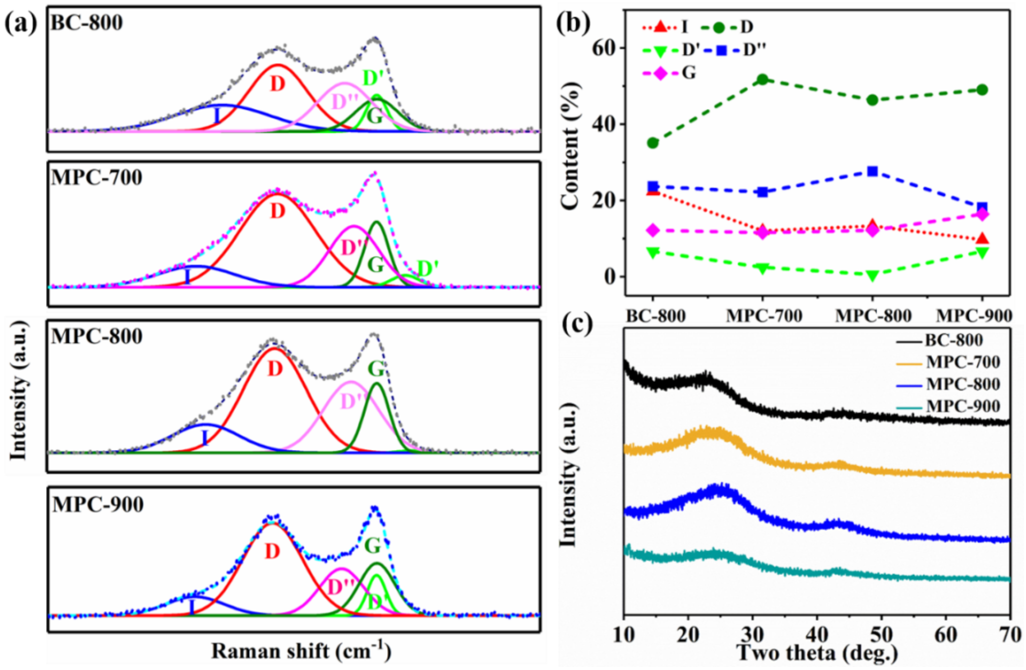

Fig. S4 (**a**) Raman spectra. (**b**) Relative structural composition derived from the fitted Raman spectra. (**c**) XRD patterns

Table S1 Characterization of pores and specific capacitance of MPC-800 and u-MPC

Sample	*C (%)*	*O (%)*	*N (%)*	*S (%)*
**BC-800**	87.4	11.2	1.4	-
**MPC-800**	86.1	13.2	0.7	-
**u-MPC**	77.8	15.8	5.2	1.2

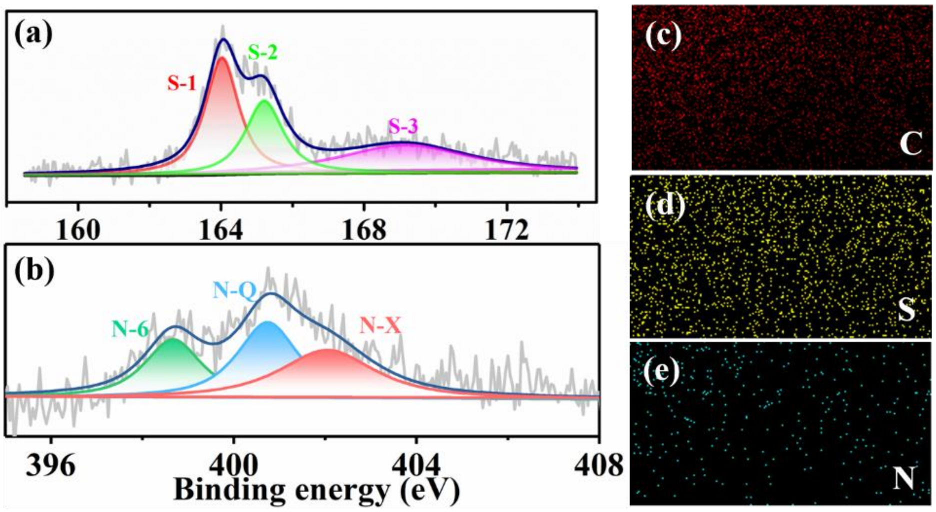

Fig. S5 High-resolution spectra of XPS spectrum (**a**) sulfur and (**b**) nitrogen. EDS images (**c**) carbon, (**d**) sulfur, and (**e**) nitrogen

Table S2 Characterization of pores and specific capacitance of MPC-800 and u-MPC

Sample	S_{BET} (m^2/g)	S_{mic} (m^2/g)	S_{ext} (m^2/g)	V_t (cm^3/g)	V_{mic} (cm^3/g)	D_{ave} (nm)
**BC-800**	676.65	565.19	111.46	0.44	0.23	2.58
**MPC-800**	1554.54	1311.35	243.20	0.65	0.52	2.36
**u-MPC**	1704.20	1470.45	233.74	0.81	0.62	2.19

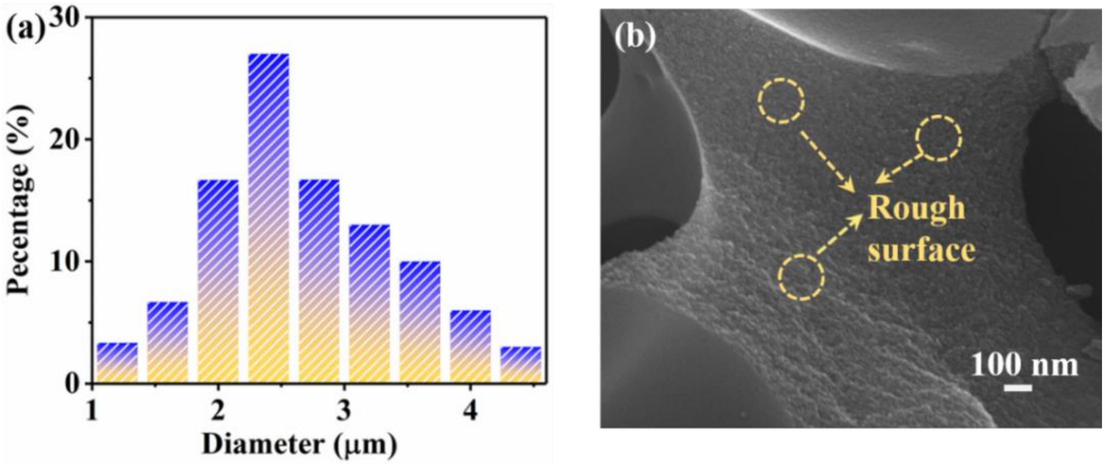

Fig. S6 (**a**) Morphology of u-MPC and particle size distribution. (**b**) Sectional morphology of u-MPC carbon particle

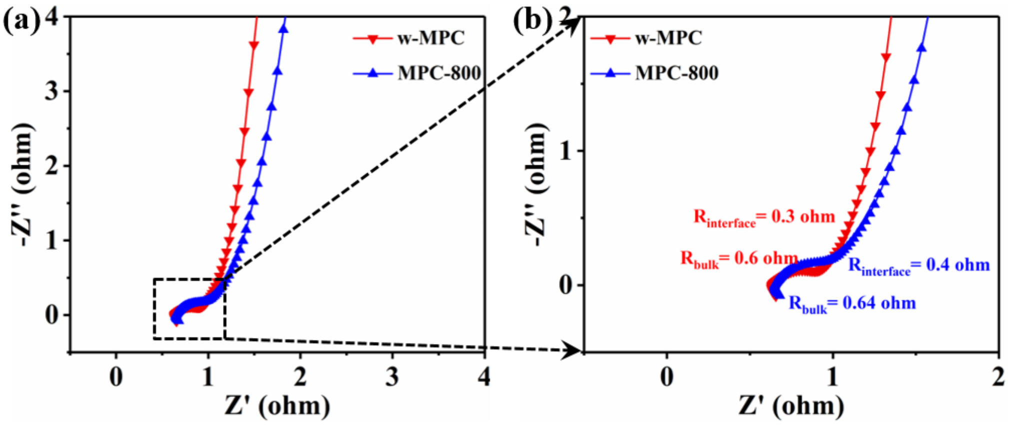

Fig. S7 (**a**) Impedance curves of u-MPC and MPC-800 and (**b**) magnified parts

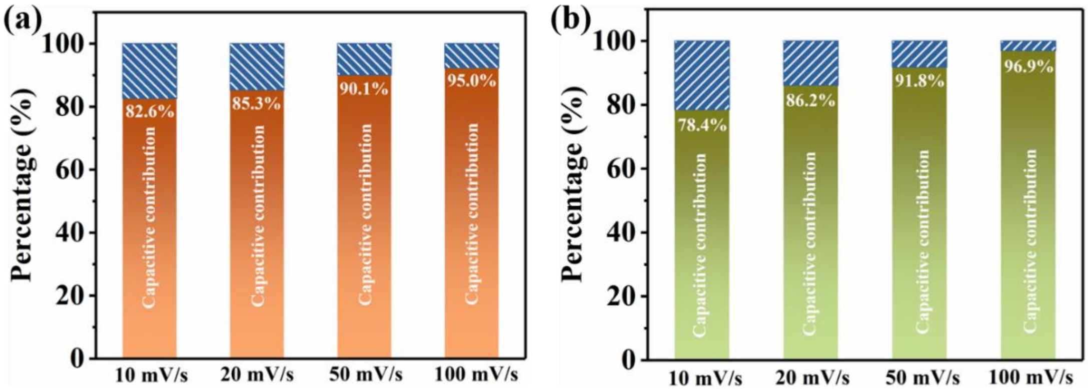

Fig. S8 Capacitive and diffusive contributions to the total stored charge of (**a**) MPC-800 and (**b**) u-MPC determined by Dunn's method

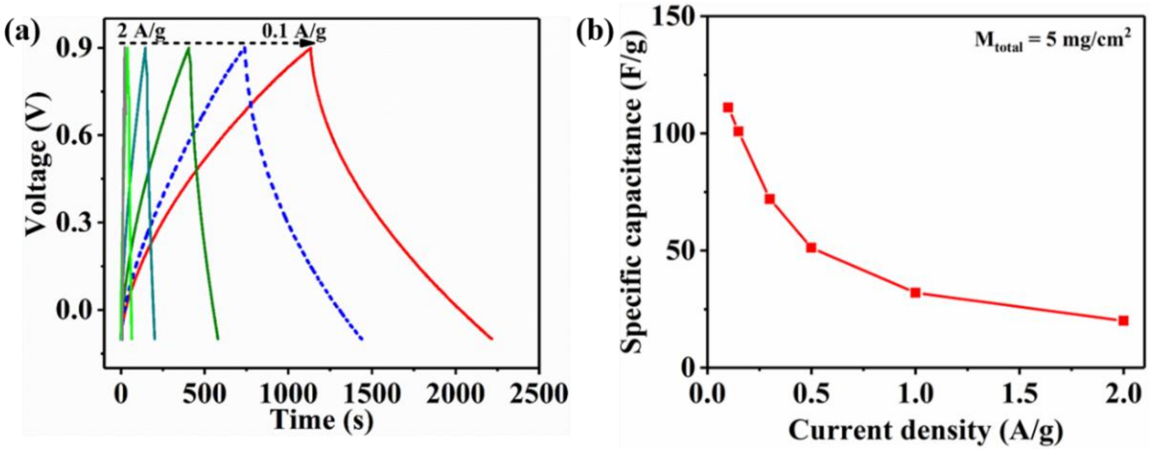

Fig. S9 (**a**) Galvanostatic charge/discharge curves of symmetric supercapacitor at various areal current density. (**b**) Specific capacitance of supercapacitor versus various current densities

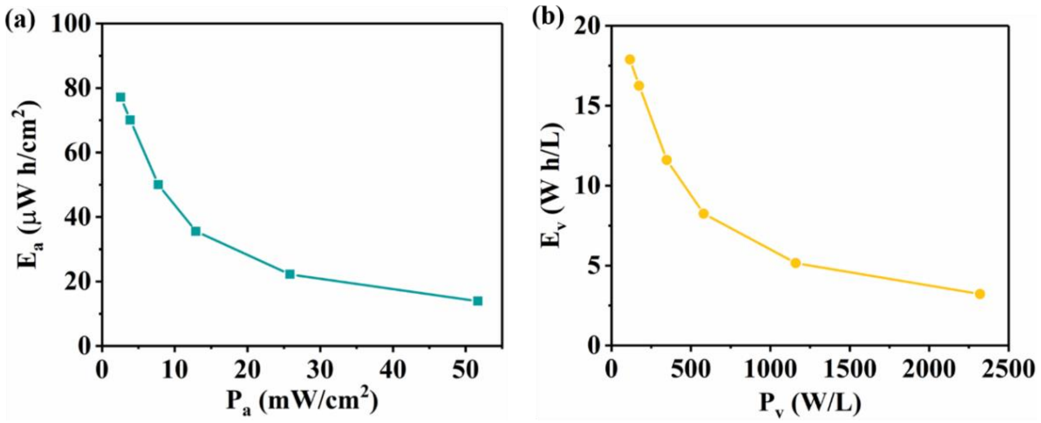

Fig. S10 (**a**) Areal energy density versus areal power densities. (**b**) Volumetric energy density versus volumetric power densities

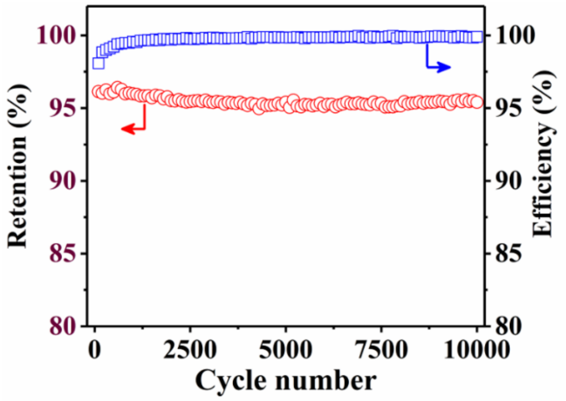

Fig. S11 Long-term cyclic stability of symmetric supercapacitor at 0.3 A g^{-1}

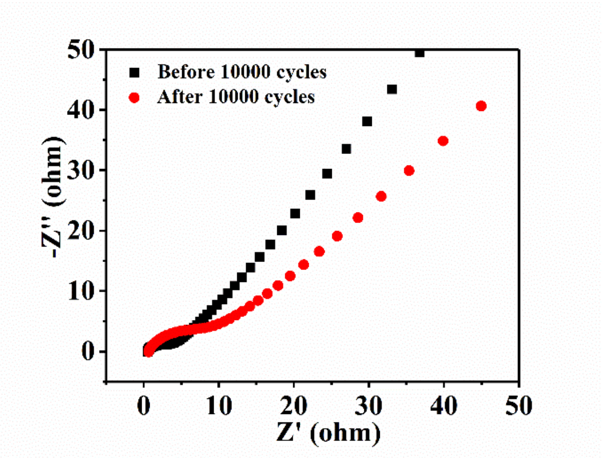

Fig. S12 Impedance curves of symmetric device before and after 10,000 cycles
